# Outsmarting trogocytosis to boost CAR NK/T cell therapy

**DOI:** 10.1186/s12943-023-01894-9

**Published:** 2023-11-16

**Authors:** Faezeh Ramezani, Ahmad Reza Panahi Meymandi, Behnia Akbari, Omid Reza Tamtaji, Hamed Mirzaei, Christine E. Brown, Hamid Reza Mirzaei

**Affiliations:** 1 Division of Medical Biotechnology, Department of Medical Laboratory Sciences, School of Paramedical Sciences, Shiraz University of Medical Sciences, Shiraz, Iran; 2 Diagnostic Laboratory Sciences and Technology Research Center, School of Paramedical Sciences, Shiraz University of Medical Sciences, Shiraz, Iran; 3 Department of Medical Immunology, School of Medicine, Tehran University of Medical Sciences, Tehran, Iran; 4 Electrophysiology Research Center, Neuroscience Institute, Tehran University of Medical Sciences, Tehran, Iran; 5 Department of Physiology, School of Medicine, Tehran University of Medical Sciences, Tehran, Iran; 6 Research Center for Biochemistry and Nutrition in Metabolic Diseases, Institute for Basic Sciences, Kashan University of Medical Sciences, Kashan, Iran; 7 Department of Hematology & Hematopoietic Cell Transplantation, City of Hope Medical Center, Duarte, CA USA; 8 Department of Immuno-Oncology, City of Hope Beckman Research Institute, Duarte, CA USA; 9 Molecular Imaging and Therapy Service, Department of Radiology, Memorial Sloan Kettering Cancer Center, New York, NY 10065 USA

**Keywords:** Trogocytosis, CAR T cells, CAR NK cells, Cancer immunotherapy

## Abstract

Chimeric antigen receptor (CAR) NK and T cell therapy are promising immunotherapeutic approaches for the treatment of cancer. However, the efficacy of CAR NK/T cell therapy is often hindered by various factors, including the phenomenon of trogocytosis, which involves the bidirectional exchange of membrane fragments between cells. In this review, we explore the role of trogocytosis in CAR NK/T cell therapy and highlight potential strategies for its modulation to improve therapeutic efficacy. We provide an in-depth analysis of trogocytosis as it relates to the fate and function of NK and T cells, focusing on its effects on cell activation, cytotoxicity, and antigen presentation. We discuss how trogocytosis can mediate transient antigen loss on cancer cells, thereby negatively affecting the effector function of CAR NK/T cells. Additionally, we address the phenomenon of fratricide and trogocytosis-associated exhaustion, which can limit the persistence and effectiveness of CAR-expressing cells. Furthermore, we explore how trogocytosis can impact CAR NK/T cell functionality, including the acquisition of target molecules and the modulation of signaling pathways. To overcome the negative effects of trogocytosis on cellular immunotherapy, we propose innovative approaches to modulate trogocytosis and augment CAR NK/T cell therapy. These strategies encompass targeting trogocytosis-related molecules, engineering CAR NK/T cells to resist trogocytosis-induced exhaustion and leveraging trogocytosis to enhance the function of CAR-expressing cells. By overcoming the limitations imposed by trogocytosis, it may be possible to unleash the full potential of CAR NK/T therapy against cancer. The knowledge and strategies presented in this review will guide future research and development, leading to improved therapeutic outcomes in the field of immunotherapy.

## Introduction

Conventional cancer treatment modalities, such as surgery, radiotherapy, and chemotherapy have long been utilized in the clinic to treat cancer [1]. Despite some progress, the tumor-free survival rate of patients following standard therapy for most tumors remains low [1]. Furthermore, surgery and radiation therapy are only beneficial for regional or localized tumors, and chemotherapy lacks selectivity in discriminating between healthy and malignant cells. Targeted immunotherapies have significantly improved clinical outcomes in cancer treatment over the last few decades [2]. CAR T cell therapy, one of the most promising cancer immunotherapies, has received regulatory approval for treatment of B cell malignancies [3]. CARs are synthetic antigen receptors that redirect the specificity and function of T cells and other immune cells to a target of interest, bypassing the need for MHC presentation [4]. Natural Killer (NK) cells are another promising cellular platform for CAR therapy because they can be isolated from various sources and safely administered regardless of donor-patient matching, thereby having the potential to significantly lower treatment costs [5].

Despite the success of CAR NK/T cell therapies, multiple barriers remain that limit their effectiveness, including intrinsic obstacles of the tumor microenvironment (TME), in particular in solid tumors [6]. The hostile TME not only limits CAR NK/T cell trafficking to the target site, but also disrupts their metabolic function, and leads to cellular exhaustion of CAR NK/T cells [7]. Additionally, some malignant cells, in both hematologic and solid tumors, restrict antigen expression to shield themselves from recognition and anticancer immune responses [8]. For instance, immunoediting and the resulting antigen loss remain a significant obstacle for curative treatment in up to 25% of leukemia patients [9]. Relapse or resistance to CAR T cell therapy is primarily due to either lack of T cell persistence or antigen loss [10]. A variety of cellular and molecular pathways can contribute to tumor low antigen density and/or antigen loss including localized loss-of-function mutations, alternative splicing, dysregulated trafficking to the cell surface, lineage switching, and trogocytosis [11, 12].

Trogocytosis is the transfer of surface molecules during cell-to-cell contact, which can occur during ‘immunological synapse’ formation. It is an active transfer of membrane components between two live cells, and any type of cell can execute trogocytosis [13, 14]. Recipient cells can receive membrane-associated proteins and acquire different cellular functions, while donor cells may lose these proteins and cellular functions. Trogocytosis can occur in both directions, allowing reciprocal transfer of membrane-associated proteins and cellular functions. Trogocytosis can occasionally mediate the transfer of plasma membranes and intracellular components across cells, which may also have an impact on cellular processes [15, 16]. The trogocytosis-transplanted membrane proteins maintain their orientation and functionality until they undergo regular turnover and get recycled [16]. In experimental settings, trogocytosis can be identified by observing moderate protein expression of the marker of interest, coupled with either low or absent gene expression of the same marker. Therefore, one possible method for detecting trogocytosis is to assess both the protein and gene expression levels of the potential trogocytic marker within cells [17].

Trogocytosis by immune cells has been recognized as an evolutionarily beneficial mechanism that can contribute to the efficiency and adaptability of the immune system [18]. This process allows immune cells to sample antigens from neighboring cells without requiring cell engulfment, and enabling rapid assessment of the antigenic profile and quick responses to potential threats [19]. By acquiring antigens from infected or abnormal cells, immune cells gain valuable insights into the presence of foreign or pathogenic molecules, enhancing immunological surveillance [20]. For instance, neutrophils can employ trogocytosis to kill antibody opsonized tumor cells through by the endocytosis of cytoplasmic fragments of the target cell, which results in cell death through a lytic process [21]. Trogocytosis also plays a critical role in antigen presentation, allowing antigen-presenting cells to capture and present antigens to T cells, and thereby initiating and regulating adaptive immune responses [22, 23]. For example, TCR engagement with MHC-presented tumor antigens can lead to trogocytosis-mediated antigen transfer. This process can enhance T cell activation and antitumor responses [24]. However, it can also lead to antigen escape if tumor cells lose antigens during trogocytosis, potentially making them less detectable to the immune system [25]. The exchange of surface receptors and molecules during trogocytosis can also influence the responsiveness and sensitivity of immune cells to certain stimuli, contributing to immunomodulation and immune system homeostasis Additionally, trogocytosis may facilitate the transfer of immunological information between immune cells, contributing to the establishment and maintenance of immunological memory [20].

The role of trogocytosis in immune cell function is context dependent. For instance, both virus-infected cells and cancer cells are engaged in trogocytosis, but the consequences are different. In virus-infected cells, trogocytosis facilitates the presentation of viral antigens to immune cells, such as T cells, triggering an immune response that leads to the eradication of infected cells by the immune system [20]. This process is beneficial in fighting viral infections. Conversely, in the cancer setting, trogocytosis can play a significant role in tumor immune evasion and immune suppression. Cancer cells may exploit trogocytosis to escape immune surveillance, leading to a detrimental effect on antitumor immune responses [12, 25]. For example, studies have shown that trogocytosis may hinder the effectiveness of CAR therapy by transferring CD19 from cancer cells to CAR T cells, resulting in the reduction of tumor antigen levels [25]. Additionally, CAR T cells that have acquired CD19 through trogocytosis can be recognized and destroyed by neighboring CAR T cells, a phenomenon known as fratricide [25]. Therefore, inhibiting trogocytosis may augment the effectiveness of CAR T cell therapy [12].

In this review, we take a deeper look into mechanisms of trogocytosis in both CAR NK cells and CAR T cells to further improve their antitumor potential in clinical applications. Understanding the role of trogocytosis in tumor-immune cell interactions will help in optimizing and enhancing the efficacy of CAR-based immunotherapies and other cancer treatments.

## The role of trogocytosis in NK cells fate and function

NK cells are a crucial component of the immune system and play a substantial role in cancer immunotherapy. NK cells identify and eradicate virus-infected cells and tumor cells by integrating signals from both activating and inhibitory receptors. This makes them crucial in early cancer cell detection and lysis. The cells can distinguish between normal cells and cancerous cells by detecting the loss of expression of MHC class I molecules or expression of stress molecules during “missing-self” or “induced-self” recognition. Once an NK cell recognizes a target cell, it produces various proinflammatory and immunosuppressive cytokines and growth factors [26]. Trogocytosis has been shown to play an important role in regulating the sensitivity of NK cells to target cells. The following list describes the molecules/receptors that can change the fate and/or function of NK cells through trogocytosis.

### Trogocytosis-mediated effects on NK Cell activation and function

NKG2D, a receptor expressed by NK cells, functions in “induced-self” recognition, a process that identifies abnormal or stressed tumor cells. NKG2D binds to altered surface molecules called NKG2D ligands (NKG2DLs), triggering NK cells to eliminate the tumor cells even in the presence of inhibitory receptors [27]. However, human cancer cells have developed several strategies to evade or develop resistance to NKG2D-mediated antitumor immunity [28, 29]. Trogocytosis is one such strategy in which NK cells acquire NKG2DLs from tumor cells through cell-to-cell contact. For example, tumor-experienced NK cells have been shown to take-up tumor-derived Rae-1, a ligand for NKG2D, resulting in their detection and killing by other tumor-naïve NK cells via the NKG2D-induced perforin pathway [27]. As a result, trogocytic transfer of NKG2DLs from cancer cells to NK cells can suppress their antitumor functions by downregulating NKG2D and increasing NK cell fratricide and death [23, 27]. Nevertheless, some researchers propose that this process might represent an intentional negative feedback mechanism aimed at regulating the hyperactivity of NK cells [23]. Furthermore, studies have indicated that NK cells have the capability to self-express NKG2DLs [30, 31]. This complicates the distinction between self-expressed NKG2DLs and those acquired through trogocytosis. To determine the origin of NKG2DLs, it is essential to assess both their protein and gene expression levels. NKG2DLs acquired through trogocytosis typically exhibit moderate protein expression but lack gene expression. However, additional investigations are imperative to refine and enhance the accuracy of trogocytosis detection methods in cells. Trogocytosis can also render murine NK cells dysfunctional by acquiring m157, the murine cytomegalovirus (MCMV)-encoded ligand for the Ly49H activating receptor [32]. When m157 is acquired and binds to the Ly49H molecule expressed by NK cells, their cytotoxic function and IFN-γ production decreases following NK cell stimulation in vitro and in vivo [32–34]. Additionally, trogocytosis of PD-1 from leukemia cells was shown to reduce NK cell antitumor immunity in patients with clonal plasma cells [35]. These examples suggest that strategies to interrupt trogocytosis may reduce NK cell fratricide and augment NK cell-mediated antitumor immunity [23].

### Trogocytosis-mediated NK Cell suppression and escape mechanisms

HLA-G is a non-classical MHC Class I molecule with restricted tissue distribution to immune-privileged regions, and suppresses the activity of immune cells, including NK cells [36]. Tumors often ectopically express HLA-G, and i*n vitro* experiments have demonstrated that expression of HLA-G makes cancer cells more resistant to cytolysis and helps them to escape from immunosurveillance [37]. Tumors may inhibit NK cytotoxicity through interaction between HLA-G and NK inhibitory receptors ILT-2 and/or KIR2DL4 [38]. Activated NK cells can indeed acquire HLA-G1 from tumor cells through trogocytosis. Through a cell-to-cell contact-dependent process, nearly all activated NK cells quickly acquire detectable levels of HLA-G1. The acquisition of HLA-G1 by NK cells decreases their proliferation and promotes a suppressive phenotype that inhibits the cytolytic function of other NK cells. In this case, trogocytosis makes the effector cells act like suppressor cells through the engagement with a molecule that is not naturally expressed by these cells [39]. Apart from non-classical MHC molecules, data has revealed that T cell immune responses can be modulated by NK trogocytosis of DC cell surface molecules. NK cells have been shown to acquire the expression of MHC II, but not CD80/CD86 costimulatory molecules from dendritic cells through trogocytosis. Since DC-dressed NK cells lack functional levels of costimulatory molecules, they can thereby suppress T cell immune responses [40].

### Trogocytosis-mediated effects on infiltration and penetration of NK cells

Tumor infiltration of immune cells is a critical factor, which is associated with immune surveillance and better clinical outcome [41]. CCR7 (C-C chemokine receptor type 7) signaling, which mediates the migration of both cancer and immune cells to the lymph nodes, is implicated in lymph node metastases and several chronic inflammatory diseases [42, 43]. Recent research has revealed that the interaction between MHC-specific, activating killer Ig-like receptors (KIRs) and their ligands, specifically the binding of KIR2DS1 to HLA-CC2 and KIR2DS4 to HLA-Cw4 or HLA-Cw6, substantially enhances the ability of NK cells to acquire CCR7 through trogocytosis [44, 45].

In addition to CCR7, trogocytosis-mediated expression of other proteins has been shown to regulate NK cell activity. One example is CD9, a protein involved in several cellular processes like proliferation and the establishment of immune cell synapse. CD9 may also play a critical role in controlling the HGSC (Tubo-ovarian high-grade serous carcinoma) tumor microenvironment. CD9 trogocytosis from tumor cells onto NK cells were shown to promote reactivation of A disintegrin and metalloprotease (ADAM) 10 and 17 which both participate in the shedding of NK ligands and NK receptors. Intratumoral CD9-expressing NK cells were shown to facilitate immune escape, particularly by reducing antitumor cytokine production and increasing pro-angiogenic IL-8. The targeting of CD9 in NK cells through either a blocking antibody or CRISPR-mediated gene deletion can restore their cytotoxicity in these settings, revealing the immune suppression-related mechanisms in HGSC [46–49].

Altogether, trogocytosis-mediated interactions between NK cells and target cells play a substantial role in shaping the function and fate of NK cells in cancer immunotherapy (Table 1). Understanding the molecular mechanisms underlying trogocytosis and its impact on NK cell behavior is critical for the development of targeted immunotherapeutic strategies. Further research is needed to explore the therapeutic potential of interrupting trogocytosis-mediated immune evasion mechanisms and to explore novel targets for enhancing NK cell-mediated antitumor responses.

**Table 1:** The Role of Trogocytosis in NK Cells Fate and Function

Trogocytic molecules/receptors	Direction	Effects on NK cells fate and/or function	References
NKG2DLs	Cancer cells to NK cells	Downregulates NKG2D and increases NK cell fratricide	[23, 27]
m157	Cancer cells to NK cells	Reduces cytotoxic function and decreases IFN-γ production	[32–34]
PD-1	Cancer cells to NK cells	Reduces NK cell-mediated antitumor immunity	[35]
HLA-G1	Cancer cells to NK cells	Promotes acquisition of suppressive function by NK cells that prevents cytotoxic function of other NK cells.	[39]
MHC II and/or CD80/CD86	Dendritic cells to NK cells	Suppress T cell-mediated immune response through the lack of functional levels of costimulatory molecules in DC-dressed NK cells	[40]
CCR7	Dendritic cells (DCs) and T-cell blasts to NK cells	Promotes migration to lymph nodes	[44] [45]
CD9	Cancer cells to NK cells	Facilitates immune escape of intratumoral CD9-dressed NK cells, particularly through reduced antitumor cytokine production and increased pro-angiogenic IL-8	[46–49]

## The role of trogocytosis in T cells fate and function

In this section, we discuss critical aspects of T cell functional activity, which can be affected by trogocytosis (Table 2).

### Trogocytosis-mediated T cell activation, antigen presentation, and signaling

The acquisition of membrane components through trogocytosis allows T cells to incorporate important molecules from antigen presenting cells (APCs), such as adhesion molecules (e.g., ICAM-1, MHC:peptide complexes) and costimulatory molecules (e.g., CD86, CD80, PD ligand 1, OX40 ligand). These molecules play a critical role in T cell activation and formation of immunological synapse [50–57]. Interestingly, various studies have indicated that trogocytosis is influenced by the cellular activation state, as activated and memory T cells show greater efficiency in trogocytosis compared to naïve T cells [51, 58–60]. The enhanced trogocytosis in activated and memory T cells could be attributed to their larger size. Their size leads to increased membrane contact with APCs, or higher avidity of the memory T cell for target antigens [60]. Trogocytosis-mediated transfer of immunological synapse molecules from APCs to T cells enables the trogocytic T cells, also known as “CD8 + T-APC”, to present antigens and control activation of responsive T cells [52, 56, 57, 61–64]. For example, tumor-specific CD8 + T cells can stimulate other cytotoxic T lymphocytes (CTLs) with the same antigen specificity by re-presenting tumor-derived antigens obtained through trogocytosis. This trogocytosis-mediated T cell-T cell immunological synapse results in effector CTL activation, as evidenced by proliferation, cytokine release, and degranulation. This phenomenon can also result in fratricide of trogocytic CD8 + T cells. Different studies have shown that trogocytosis is not limited to conventional T cells, but it has also been observed in γσ T cells, a subset of T cells that exhibit direct cytotoxicity against various cancer cells and modulate antitumor cytokines [65]. Vγ9Vσ2-T cells, a subset of γσ T cells, can function as antigen-presenting cells and present glycolipid antigens to invariant natural killer T (iNKT) cells through the uptake of membrane fragments containing CD1d from target cells. Trogocytosis in Vγ9Vσ2-T cells occurs rapidly after T cell receptor engagement and is pAg-specific and time-dependent [66].

Additionally, trogocytosis-mediated signaling has been found to affect intracellular signaling, differentiation of effector T cells, and cytokine production, which can shape immune responses [67]. The intercellular transfer of APC-derived membrane fragments including MHC:peptide complexes to T cells through trogocytosis leads to persistent signaling, characterized by prolonged T cell receptor proximal and distal signaling cascades, as determined by phosphorylation of ZAP-70 and ERK 1/2 respectively. This persistent signaling can impact T cell fate by either stimulating activation-induced cell death (AICD) or clonal exhaustion, or by altering T cell effector functions and cytokine production [67]. Moreover, trogocytosis-mediated signaling was shown to affect the differentiation of effector T cell and cytokine production and consequently may play a role in preserving, augmenting, or driving, Th2-mediated immune responses. More specifically, trogocytosis-mediated signaling has been shown to induce Th1 to Th2 polarization when Th1-polarized trogocytosed T cells start to produce Th2-associated cytokines in vitro [68].

### Trogocytosis-mediated Immune suppression and escape mechanisms in T cells

In some cases, trogocytosis can contribute to the escape of cancer cells from immune surveillance. Cancer cells can suppress the activity of APCs through trogocytosis, hindering immune responses. For example, transfer of CD137, a TNF receptor family member, from cancer cells to APCs via trogocytosis resulted in the internalization of the CD137-CD137L complex in vitro. This internalization subsequently hindered the ability of APCs to provide costimulation to T cells, consequently reducing the secretion of proinflammatory cytokines by T cells [69, 70]. A comprehensive examination of costimulatory receptor expression within the tumor microenvironment and the impact of trogocytosis remains an important area for future investigation.

Additionally, cancer cells can employ trogocytosis in other ways to evade immune surveillance. As mentioned earlier, the tolerogenic nonclassic HLA-G, acquired from tumor cells can convert effector T cells into induced regulatory T cells (iTreg). This transformation subsequently hampers the function of other T cells. The acquisition of HLA-G through trogocytosis represents a potential tumor escape strategy [71]. Furthermore, recent data have shown that Treg cells can acquire CD80/CD86 molecules from APCs through trogocytosis in a CTLA-4 dependent manner, thereby promoting the suppressive function of APCs and Tregs [72]. However, Treg-mediated immune suppression, including the transfer of costimulatory molecules, can be impeded by immune checkpoint inhibitors toward CTLA-4 and PD-1/PD-L1, which are used in cancer immunotherapy [73]. Trogocytosis can also contribute to immunological control and modulation of T cell responses. Low-avidity CTLs have been shown to inhibit high-avidity CTLs in an antigen-specific manner through trogocytosis. Low-avidity CTLs strip cognate peptide-MHC complexes from target cells without killing them, leading to the suppression of cytotoxic activity of high-avidity CTLs [74]. This unique mechanism of immunological control has implications for vaccine development and may partially explain the limited success observed in certain cancer peptide vaccination trials [74]. Lastly, data have shown that neutrophils in the tumor microenvironment can acquire a suppressor function and modulate T cell-mediated immune responses through trogocytosis. Studies have shown that neutrophils and T cells co-cultured in the presence of ascites fluid from ovarian cancer patients establish stable interactions, and neutrophils acquire fragments of T cell membrane through trogocytosis. Such trogocytosis-mediated interaction can lead to neutrophil-driven immune suppression, which eventually induces suppression in T cells close to unstimulated levels [75].

**Table 2:** The Role of Trogocytosis in T Cells Fate and Function

Trogocytic molecules/receptors	Direction	Effects on T cells fate and/or function	References
Costimulatory molecules	APCs to T cells	Controls T cell activation	[52, 56, 57, 61–64]
CD86	APCs to T cells	Modifies the function and phenotype of immune effector cells, resulting in the development of regulatory capabilities that inhibit the proliferation of other T cells	[71]
CD80/CD86	APCs to Treg cells	Promotes APCs and Tregs suppressive functions	[72]
CD1d	Target cells to γσ T cells	Promotes glycolipid antigen presentation to invariant natural killer T cells (iNKT)	[66]
MHC:peptide complexes	APCs to T cells	Reduce T cell effector functions and cytokine production by promoting activation-induced cell death or clonal exhaustion	[67]
CD137	Cancer cells to APCs	Reduces T cell costimulation capacity and proinflammatory cytokine release	[69, 70]
HLA-G	Cancer cells to T cells	Converts effector T cells into iTregs	[71]

## Trogocytosis in CAR-expressing cells

**Fig. 1:**
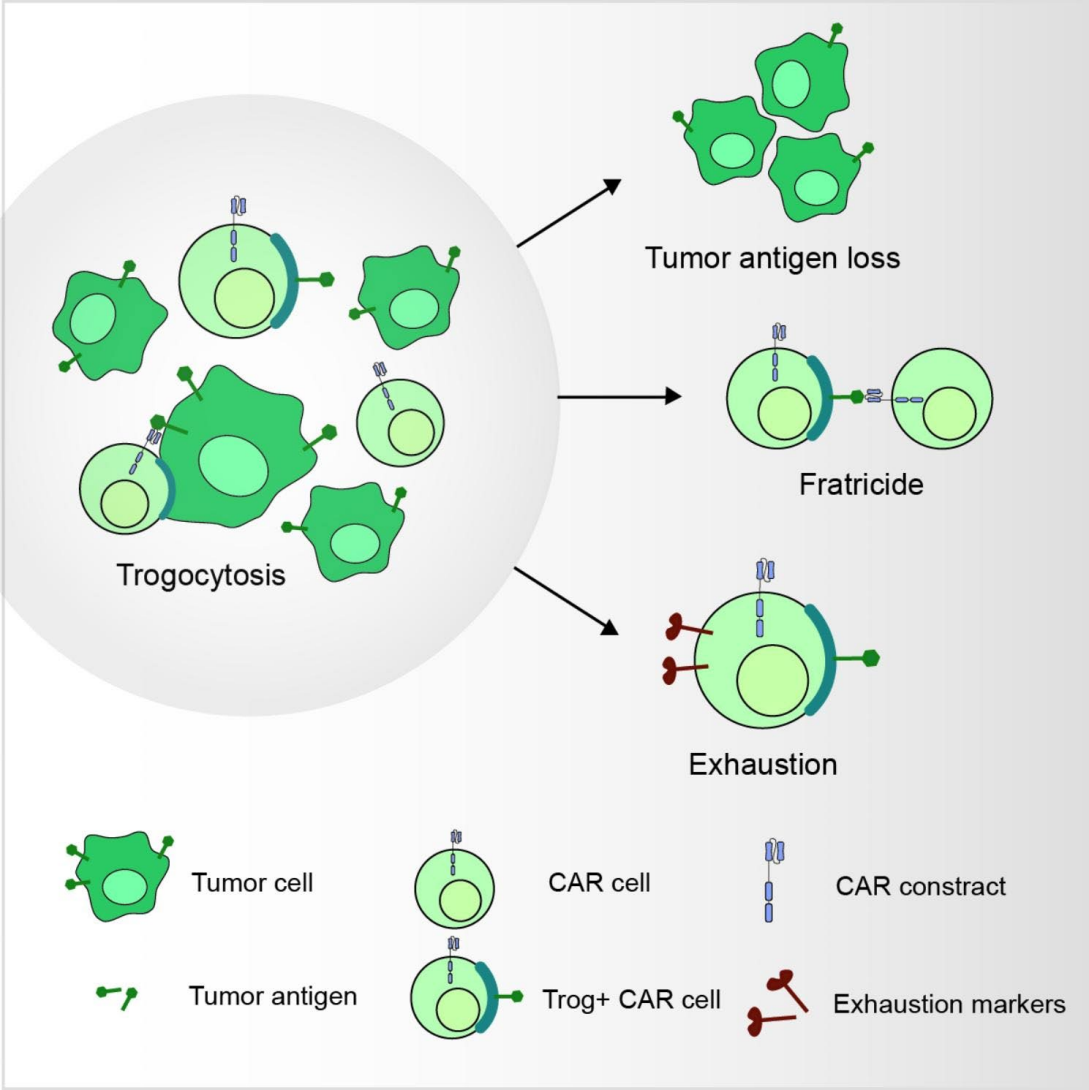
Trogocytosis alter the efficacy and antitumoral functions of CAR-expressing cells. Trogocytosis can reduce tumor antigen density and promote fratricide or exhaustion of CAR-expressing cells

As described above, trogocytosis, a phenomenon extensively studied in NK cells and T cells, has been exhibited to have both positive and negative effects on the function of accepting cells [18, 22]. Cellular and molecular characteristics of the target cell and its microenvironment determine the functional outcomes of trogocytosis in T cells [76]. Recent reports have shown that cancer cells undergo trogocytosis when exposed to CAR-expressing cells both in vitro and in vivo. This poses a challenge for CAR therapies as it leads to antigen downregulation and fratricidal destruction of CAR-expressing cells [17, 25, 76, 77]. The regulation of effector trogocytosis and its alteration during tumor growth remains to be completely understood. Current evidence suggests that tumor-derived factors (TDFs) promote trogocytosis between CAR-expressing and tumor cells via activating transcription factor-3 (ATF3) [76]. ATF3 is an early stress response gene that is activated by many factors in TME, including nutrient deprivation and hypoxia [78]. TDF-induced ATF3 in CAR T cells reduces cholesterol 25-hydroxylase (CH25H) expression, which catalyzes monooxygenation of cholesterol into 25-hydroxycholesterol (25HC) [76, 79]. The latter molecule influences the fluidity and ability of lipid membranes to undergo fusion during trogocytosis [80–82], which is necessary for incorporating membrane molecules of malignant cells into CAR T cells [83, 84]. Perturbation of the ATF3-CH25H pathway in intratumoral nonCAR- or CAR-expressing cytotoxic T lymphocytes promotes effector trogocytosis, compromising cell viability and antitumor activity [76]. This section highlights how trogocytosis influences the efficacy and antitumoral functions of CAR-expressing cells and specifically leads to tumor cell antigen loss, fratricide, and CAR cell exhaustion (Fig. 1).

### Trogocytosis-induced tumor antigen heterogeneity

Downregulation or loss of tumor antigens on the target cells has been observed in multiple tumor models and clinical samples after CAR T cell therapy. Immunological pressure and trogocytosis are two mechanisms which contribute to tumor antigen loss or downregulation. Mechanistically, CAR targeting of tumor cells promotes trogocytosis, resulting in reduced antigen density on target cancer cells to a level that can elude CAR recognition and thereby foster CAR T cell resistance [14, 17, 25, 77]. While CAR NK has also been shown to undergo trogocytosis [17], CAR T cells are more extensively studied in this context [14, 25, 77]. In addition, the immunological pressure conferred by CAR T cells can further promote the selection and outgrowth of antigen-low tumor variants [8].

CAR T cell therapy is currently becoming standard of care for advanced multiple myeloma (MM) [85]. Most therapeutic approaches currently aim to target B cell maturation antigen (BCMA), which is essential for preserving the phenotype of tumor cells and is primarily expressed in B and plasma cells [86, 87]. However, anti-BCMA CAR T cell therapy has a limited duration of response, with eventual relapse in multiple myeloma [88]. The reason behind this therapeutic failure is not well understood, but likely involves several mechanisms, including trogocytosis. It has recently been discovered that the BCMA loss on target cells following CAR T cell-myeloma cell contact is, in part, due to BCMA internalization and trogocytosis by CAR T cells [77]. This phenomenon leads to a decrease in tumor antigen density and provides an opportunity for cancer cells to escape from the immune system. In the context of solid cancers, mesothelin (MSLN), has appeared as a favorable target for CAR T cell therapy owing to its expression pattern [89]. Like multiple myeloma, trogocytosis-mediated surface antigen loss poses a significant barrier to effective CAR T cell therapy of MSLN-positive solid tumors, as expression pattern of MSLN is naturally heterogeneous and trogocytosis may additionally downregulate its expression [14].

Other studies have also indicated that receptor-ligand interactions or CAR signaling itself can trigger trogocytosis in both NK and T cells. Interestingly, anti-CD19 CAR T and CAR NK cells have been shown to acquire CD19 and, to a lesser extent, other markers associated with B cells, such as CD22 and CD20, through the process of trogocytosis [17, 22, 25]. The extent of trogocytosis was associated with antigen availability, indicating that high-density targets increase the risk of trogocytosis-mediated CAR T cell dysfunction [14]. These findings may provide insights into the high relapse rates observed in patients with lymphoid malignancies treated with CAR NK cells [90]. There is also a possibility that tumor cells can acquire CAR molecules on their surface through trogocytosis. While direct evidence remains lacking, these trogocytosed CAR molecules may bind in cis to the cognate epitopes on the surface of target cells, masking them from recognition by CAR NK/T cells. Masking of tumor antigen and thereby conferring resistance to CAR T cells has been described elsewhere, however, it was not attributed to trogocytosis [90]. Therefore, further studies are needed to thoroughly investigate the potential for CAR acquisition via trogocytosis, followed by tumor antigen masking, and its potential implication for resistance to CAR NK/T therapy. Overall, surface antigen density directly influences CAR functionality, and downregulation of surface antigen can lead to tumor escape.

In summary, CAR activation and immunological synapse formation can potentially induce trogocytosis, resulting in a decrease in antigen density on target cancer cells. Antigen density can be constitutively low or transiently lowered after CAR T cell-mediated trogocytosis. In either scenario, insufficient reactivity against these cells with low antigen density can lead to outgrowth and survival of antigen-low variants. Such antigen-low variants can potentially make tumor cells more resistant to CAR T cell-mediated cytotoxicity even in the presence of long-lasting CAR T cells, likely due to escape of antigen-low variants below the CAR recognition threshold. However, further research is required to fully understand trogocytosis-mediated antigen downregulation and other still unexplored escape mechanisms following CAR T cell therapy.

### Trogocytosis-induced fratricide

Fratricide, the cytotoxicity of CAR-expressing cells against other CAR-expressing cells, has been demonstrated to be enhanced by trogocytosis-mediated transfer of CAR target antigens to CAR-expressing cells [14, 17]. Recent studies have shown that the high-affinity CD19 binding domain FMC63-composed CAR T cells can acquire CD19 from malignant B cells and add it to their own plasma membrane [25]. Moreover, CD19-expressing CAR T cells can be targeted by CD19-specific CAR T cells, implying that fratricide may also happen spontaneously after CAR T cells capture CD19 through trogocytosis [25]. Additionally, the expression and acquisition of MSLN by anti-mesothelin CAR (mesoCAR) T cells composed of CD28 or 4-1BB costimulatory domains poses a risk for cytolysis of CAR T cells expressing MSLN by MSLN-directed CAR T cell [14]. Fratricidal cytotoxicity may explain the reduced viability and proliferation observed in trogocytosis-positive (trog+) CAR T cells compared to trogocytosis-negative (trog-) CAR T cells [14]. However, additional investigations are needed to better understand the various aspects of fratricidal killing between CAR T cells.

### Trogocytosis-induced CAR cell exhaustion and hyporesponsiveness

Chronic antigenic stimulation by cancer cells is well recognized to induce exhaustion of immune effector cells [91–93]. CAR-mediated trogocytosis weakens the antitumor function of CAR cells by redirecting their effector function against other CAR-expressing cells, leading to fratricide and CAR cell exhaustion [17]. Trogocytic MSLN-positive (MSLN+) mesoCAR T cells exhibit elevated expression levels of the inhibitory molecules LAG-3, PD-1, and/or TIM-3 compared to MSLN-negative (MSLN-) mesoCAR T cells [14]. This indicates that trogocytic mesoCAR T cells might be functionally exhausted or even dysfunctional compared to their non-trogocytic counterparts [14]. This suggests that trogocytic mesoCAR T cells might be functionally exhausted or even dysfunctional compared to their non-trogocytic counterparts [14]. As mentioned earlier, the loss of CH25H in the TME promotes trogocytosis and impairs the activity and viability of antigen experienced CTLs [76]. Moreover, under these circumstances, *Ch25h-null* CAR T cells exhibiting increased trogocytosis display greater signs of exhaustion, increased apoptosis, and attenuated antitumor immune responses [76]. Importantly, the inhibition of effector trogocytosis in ATF3-ablated CAR T cells has shown reduced exhaustion, apoptosis, and improved killing of antigen-expressing malignant cells [76]. These findings suggest a significant function for the ATF3-CH25H pathway in the regulation of the degree of trogocytosis, activity and viability of CAR T cells in vitro and in vivo [76].

Interestingly, recent data have revealed that antigen-mediated self-engagement can drive exhaustion of CAR NK cells. Studies suggest that trogocytosis of the antigen by CAR NK cells that escape fratricide can lead to functional exhaustion via chronic antigen-mediated CAR activation [17]. Recent observations have demonstrated trogocytosis as a mechanism of CAR NK cell dysfunction, with CARs composed of different intracellular signaling domains and targeting distinct cancer antigens [17]. The acquisition of the cognate tumor antigen by CAR NK cells drives self-recognition and chronic CAR activation, but also decreases NK cell activity by promoting fratricide and hyporesponsiveness [17]. Considering the limited number of CAR constructs studied, these data collectively indicate that trogocytosis-mediated antigen acquisition is linked to functional exhaustion and the failure of CAR-expressing cells to control the tumor. To gain a more comprehensive understanding, further studies with additional CAR constructs are necessary to determine the role of the CAR construct design and its antigenic specificity in inducing trogocytosis and exhaustion of CAR-expressing cells.

## Modulation of trogocytosis to enhance efficacy of CAR-expressing cells in cancer

**Fig. 2:**
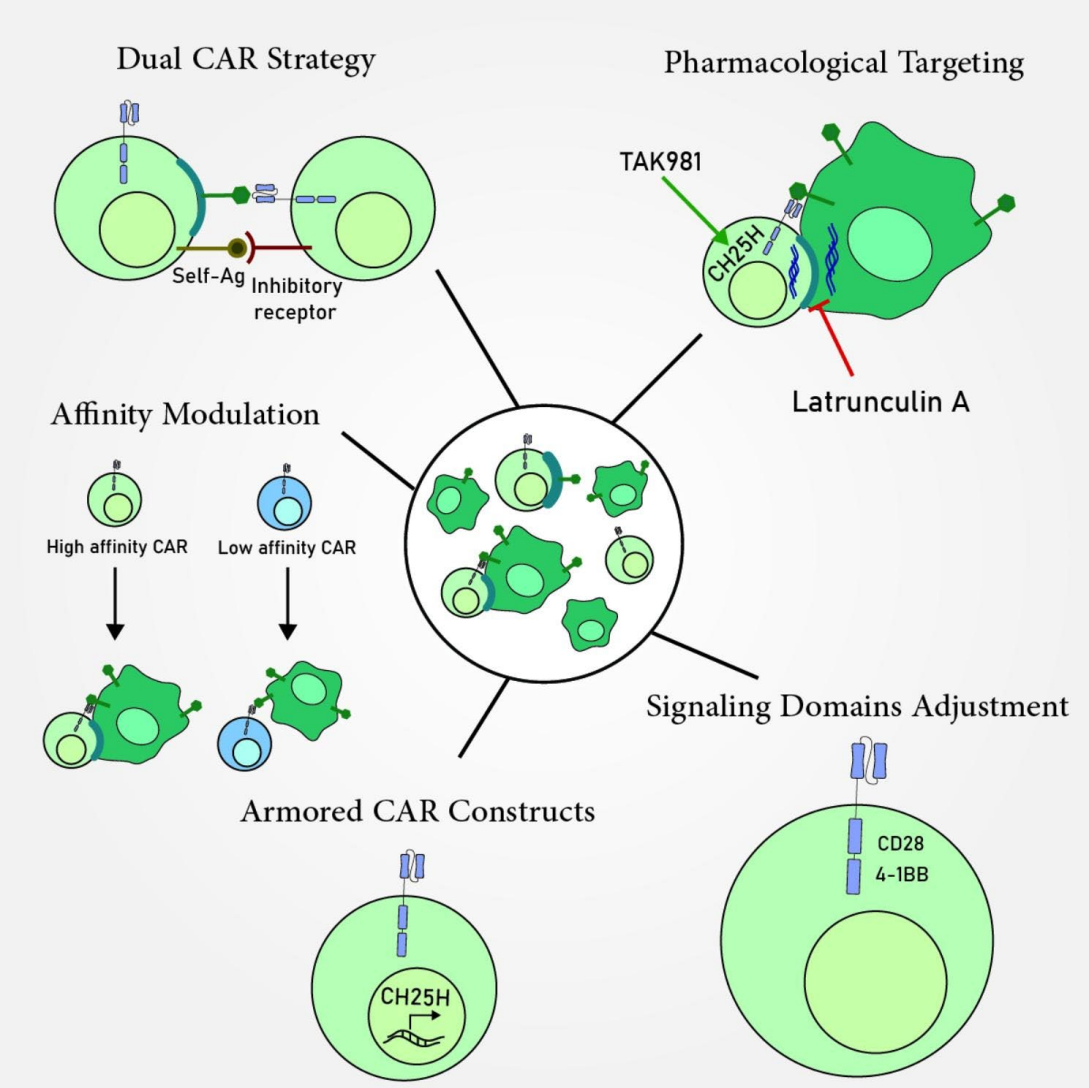
Potential approaches to enhance CAR-Cell antitumoral functions by modulating trogocytosis. Several strategies can be employed to boost antitumoral functions of these cells, including pharmacological targeting, dual CAR strategy, affinity modulation of CARs, implementing armored CAR constructs, and adjusting the signaling domains

Strategies to overcome trogocytosis-induced antigen loss, fratricide, and CAR cell exhaustion have the potential to improve CAR cell efficacy and tumor clearance. Currently, there are no specific therapeutic strategies to regulate trogocytosis. This section describes potential approaches to enhance CAR cell antitumoral functions by modulating trogocytosis (Fig. 2).

### Pharmacological targeting

#### Blockers of actin polymerization

The exchange of membrane fragments between cells during trogocytosis relies on actin remodeling [14]. Pre-treating CAR-NK cells with Latrunculin A (LatA), an inhibitor of F-actin that inhibits the formation of immunologic synapses [94], has been shown to prevent the transfer of CD19 molecule from Raji cells to anti-CD19 CAR-NK cells, highlighting the importance of immunological synapse formation in promoting trogocytosis [17]. In line with previous findings, LatA treatment of CTLs has been demonstrated to inhibit trogocytosis [25]. Additionally, pre-treatment of MSLN + ovarian cancer cell line (OVCAR-3) with LatA prior co-culture to mesoCAR T cells containing CD28 or 4-1BB costimulatory domains resulted in a 50% reduction in the frequency of MSLN + mesoCAR T cells, indicating the actin remodeling-dependent trogocytosis contributes to transfer of MSLN from target tumor cells to mesoCAR T cells [14]. Although actin polymerization blockers effectively inhibit trogocytosis in vitro, their clinical use is limited due by their toxicity to human cells. Moreover, these drugs can hinder productive immunological synapse formation, potentially compromising the cytotoxic functions of CAR NK/T cells. As a result, the application of actin polymerization blockers as a strategy to modulate trogocytosis has limited clinical applicability. Further research is needed to develop clinically relevant compounds that can more specifically target trogocytosis, while minimizing toxicity.

#### Sumoylation inhibitors

Studies have shown that intratumoral T cells experience a partial loss of CH25H, leading to enhanced trogocytosis characterized by poor survival and hypofunctionality of CAR T cells [76]. CH25H can directly block fusion of lipid membranes [80–82], which may explain its ability to disrupt trogocytosis. Boosting CH25H expression could potentially enhance the efficacy of CAR NK/T cell therapy through reduction of trogocytosis [76].

Sumoylation inhibitors offer a pharmacological approach to suppress effector trogocytosis. These inhibitors interfere with ATF3-driven downregulation of CH25H, thereby restoring CH25H expression in intratumoral cytotoxic T lymphocytes. ATF3 directly inhibits CH25H transcription, and sumoylation is often associated with transcriptional repression. Disrupting the sumoylation pathway has been reported to activate innate and adaptive immunity. Pharmacological restoration of CH25H expression using the sumoylation inhibitor TAK981 in CTLs has been shown to reduce trogocytosis, suppress tumor growth, and improve the efficacy of immunotherapies [76]. TAK981 is presently being examined in various cancer clinical trials, including NCT04381650, NCT04074330, NCT03648372, and NCT04776018. While promising, it is essential to carefully manage the clinical implications and toxicities associated with the continuous use of sumoylation inhibitors due to their impact on numerous cellular processes. Moreover, considering the importance of ATF3 as a key transcription factor that regulates various crucial genes involved in the immune response, cancer development, and glucose homeostasis [95], it will be crucial to selectively target trogocytosis in CTLs.

### Dual CAR strategy

NK cell cytotoxicity is regulated by an elegant balance between inhibitory and activating signals [96]. To address the challenge of fratricide and hyporesponsiveness caused by trogocytic tumor antigen-expressing NK cells (trog + NK cells), Li et al. have developed a novel dual CAR strategy [17]. This strategy fused an activating CAR (aCAR), which targets a specific tumor antigen like ROR1 or CD19, with an inhibitory CAR (iCAR) against a self-antigen found in NK cells (e.g., CS1) [17]. The iCAR employed the intracellular signaling domain derived from KIR2DL1, which is a type of killer cell immunoglobulin-like receptor (KIR). This domain conveys an inhibitory signal through its immune tyrosine-based inhibitory motif (ITIM) once it binds to its ligand [17]. Through harnessing the natural biology of NK cells, this iCAR averted on-target/off-tumor activation of aCARs by trog + NK cells whilst preserving on-target antitumor activity [17]. In various cancer models, NK cells co-expressing both CARs transmitted a “don’t-kill-me” signal upon engaging with trog + NK cells. This vibrant modulation of CAR signaling effectively prevents aCAR-mediated fratricide and hyporesponsiveness, thereby enhancing the in vivo persistence and antitumor response of adoptive NK cell therapies [17]. Additionally, this dual CAR strategy has the potential to reduce CAR-mediated NK cell activation toward noncancerous cells expressing the target antigen [17]. In summary, the dual CAR strategy represents a significant advancement in adoptive NK cell therapy. By combining an inhibitory CAR with an activating CAR, this approach harnesses the natural regulatory mechanisms of NK cells to improve therapy effectiveness and reduce unwanted off-target effects.

### Affinity modulation

Studies have shown that trogocytosis is associated with the avidity of TCR-peptide/MHC complexes, suggesting that a similar relationship may exist with CAR affinity and CAR T cell-mediated trogocytosis [74, 97]. Thus, lowering the antigen-specific affinity of the CAR could limit trogocytosis while maintaining efficacy. Notably, low-affinity CARs have been reported to exhibit increased efficacy in preventing tumor escape [25, 97–99]. Recent studies have shown that low-affinity anti-CD229 and anti-CD19 CAR T cells exhibited comparable target cell killing capacity, proliferation, and IFN-γ when compared to their high-affinity counterparts. Importantly, low-affinity CAR constructs significantly increased the levels of target antigen on cancer cells at the end of in vitro co-cultures, indicating reduced antigen loss [97]. It was further shown that high-affinity CAR T cells acquired cell membrane fragments from target cancer cells during co-culture, a hallmark of trogocytosis, while low-affinity CAR T cells exhibited significantly decreased membrane transfer [97]. These findings suggest that trogocytosis can be significantly limited by lowering CAR affinity, while still preserving antitumor function and clinical efficacy. In mouse models and clinical trials, low-affinity anti-CD19 CAR T cells have shown improved persistence compared to high-affinity anti-CD19 CAR T cells [100]. Possible explanations for this finding include more physiological receptor signaling may prevent activation-induced cell death or early exhaustion, as well as induction of T cells with self-renewal ability (i.e., stem-like T cells) in low-affinity CAR T cells [101, 102]. In addition to reduced trogocytosis, low-affinity CAR binding domains may provide other benefits such as increased selectivity and reduced exhaustion [97, 103, 104]. Although discovery of low-affinity CAR constructs with similar antitumor activity may not always be feasible, trogocytosis should be carefully assessed in the development of novel CAR constructs.

### Armored CAR constructs

To overcome the downregulation of CH25H in intratumoral CTLs, Lu et al. have redesigned formerly described anti-CD19 and anti-meso CARs to co-express CH25H [76, 105]. A comparison between CTLs carrying conventional CARs and CARs armored with CH25H showed that re-expression of CH25H significantly hampered trogocytosis, increased viability and tumoricidal activity of CAR T cells, and reduced exhaustion [76]. Furthermore, CH25H-expressing CAR T cells exhibited greater therapeutic efficacy against tumors, improved infiltration, and lower expression levels of exhaustion markers and apoptosis in vivo [76]. These findings suggest that overexpression of CH25H in CAR T cells make them more resistant to trogocytosis, related exhaustion, and cell death, while enhancing their antitumor function and therapeutic efficacy.

### Signaling domains modification

An alternative approach to address trogocytosis-mediated antigen loss involves adjustment of the signaling domain of the CAR as trogocytosis might potentially impact CARs with 4-1BB or CD28 signaling domains in distinct ways [25]. 4-1BB and CD28 are commonly exploited as co-stimulatory domains that affect CAR T cell persistence, functionality, and cytolytic capacity [106–108]. In a preclinical study, mice bearing SKOV3 tumors and treated with CD28-based mesoCAR T cells exhibited a prolonged survival, while those treated with 4-1BB-containing mesoCAR T cells showed long-lasting remissions in some cases [109]. Notably, both CD28 and 4-1BB mesoCAR T cells acquired MSLN antigen through trogocytosis from SKOV3 cells, leading to a subsequent loss of MSLN surface expression on the cancer cells. However, 4-1BB mesoCAR T cells significantly mediated more trogocytosis 10–25 days post-CAR therapy compared to CD28 mesoCAR T cells, and a similar trend was seen ≥ 31 days after treatment [109]. Additionally, fratricide killing of trogocytic MSLN + T cells by mesoCAR T cells was also observed [14]. Further studies are required to determine whether specific modifications to different domains of CAR constructs can diminish trogocytosis-mediated antigen loss and/or fratricide, thus improving the therapeutic efficacy of CAR-NK and CAR-T cells.

Overall, modulating trogocytosis holds great promise for enhancing the effectiveness of CAR cell-based therapies in cancer. Pharmacological strategies such as actin polymerization blockers and sumoylation inhibitors may limit antigen loss and improve the efficiency of CAR-based therapies. Furthermore, optimizing CAR design through affinity modulation, armored constructs, and modified signaling domains may reduce exhaustion, minimize trogocytosis-mediated antigen loss, and maximize the therapeutic efficacy of CAR NK/T therapies. However, these approaches require careful consideration of their clinical implications, including addressing metabolic changes and potential toxicities associated with trogocytosis modulation. Through ongoing research and development, trogocytosis modulation may become a valuable and successful therapeutic strategy for improving outcomes in CAR cell-based therapies for cancer patients.

## Discussion

This review comprehensively examined the implications of trogocytosis between CAR-expressing cells and tumor cells and proposed potential strategies to enhance the antitumoral function of CAR-expressing cells by modulating trogocytosis. Antigen loss on target tumor cells due to trogocytosis following exposure to CAR T cells is typically transient and reversible [25]. However, these events offer cancer cells a window of opportunity to evade future killing through various mechanisms, including immunoediting and loss of antigen expression [9], downregulation of MHC-I [110], production of immune-suppressive substances and ligands (such as adenosine, PD-L1, or cancer-derived extracellular vesicles) [111–113], among others. By limiting trogocytosis in CAR T cells, we can potentially close or truncate this temporal window and potentially avert resistance to adoptive immune cell therapies.

In addition to diminishing antigen density on malignant cells, trogocytosis also affects the function of CAR T cells themselves. CAR T cells that engage in trogocytosis without successfully eliminating malignant cells face several consequences. These include fratricide by other CAR T cells and trogocytosis-associated exhaustion [25]. As the antigens targeted by CARs can serve as critical signaling molecules in cancer cells (e.g., mesothelin in solid tumors or BCMA in plasma cells) [114, 115], the capture of target-associated molecules by NK or T cells via trogocytosis may potentially modify the cellular function of CAR-expressing cells. Therefore, future investigations should focus on the signaling pathways triggered by trogocytosis-acquired molecules in CAR cells and determine if this factor should be considered when designing CAR therapies. Furthermore, it is crucial to conduct further studies to elucidate how trogocytosis influences the killing capabilities of CAR T cells in conjunction with target malignant cells.

A comprehensive understanding of trogocytosis and its effects on both CAR-expressing cells and tumor cells is imperative for optimizing the therapeutic efficacy of CAR-based immunotherapies. By developing approaches that modulate trogocytosis, we can potentially overcome the challenges posed by antigen loss, immune escape, and CAR T cell dysfunction. These strategies may include pharmacological interventions, affinity modulation of CARs, implementation of armored CAR constructs, and adjustment of signaling domains. Continued research in these areas will contribute to the advancement of trogocytosis modulation as a valuable therapeutic strategy in cancer treatment.

## Data Availability

Not applicable.

## List of abbreviations

ADAM: A Disintegrin and Metalloprotease
CAR: Chimeric Antigen Receptor
CCR7: C-C Chemokine Receptor Type 7
CD9: Cluster of Differentiation 9
CRISPR: Clustered Regularly Interspaced Short Palindromic Repeats
DC: Dendritic Cells
HGSC: Tubo-Ovarian High-Grade Serous Carcinoma
HLA-G: Human Leukocyte Antigen-G
IFN-γ: Interferon-gamma
ILT-2: Immunoglobulin-Like Transcript 2
KIR2DL4: Killer Immunoglobulin-Like Receptor 2DL4
KIRs: Killer Immunoglobulin-Like Receptors
MCMV: Murine Cytomegalovirus
MHC: Major Histocompatibility Complex
NKG2D: Natural Killer Group 2D
NKG2DLs: NKG2D ligands
NK cells: Natural Killer cells
TME: Tumor Microenvironment
